# *Chordin* Is a Modifier of *Tbx1* for the Craniofacial Malformations of 22q11 Deletion Syndrome Phenotypes in Mouse

**DOI:** 10.1371/journal.pgen.1000395

**Published:** 2009-02-27

**Authors:** Murim Choi, John Klingensmith

**Affiliations:** Department of Cell Biology, Duke University Medical Center, Durham, North Carolina, United States of America; Albert Einstein College of Medicine, United States of America

## Abstract

Point mutations in *TBX1* can recapitulate many of the structural defects of 22q11 deletion syndromes (22q11DS), usually associated with a chromosomal deletion at 22q1.2. 22q11DS often includes specific cardiac and pharyngeal organ anomalies, but the presence of characteristic craniofacial defects is highly variable. Even among family members with a single *TBX1* point mutation but no cytological deletion, cleft palate and low-set ears may or may not be present. In theory, such differences could depend on an unidentified, second-site lesion that modifies the craniofacial consequences of *TBX1* deficiency. We present evidence for such a locus in a mouse model. Null mutations of *chordin* have been reported to cause severe defects recapitulating 22q11DS, which we show are highly dependent on genetic background. In an inbred strain in which *chordin^−/−^* is fully penetrant, we found a closely linked, strong modifier—a mutation in a *Tbx1* intron causing severe splicing defects. Without it, lack of *chordin* results in a low penetrance of mandibular hypoplasia but no cardiac or thoracic organ malformations. This hypomorphic *Tbx1* allele per se results in defects resembling 22q11DS but with a low penetrance of hallmark craniofacial malformations, unless *chordin* is mutant. Thus, *chordin* is a modifier for the craniofacial anomalies of *Tbx1* mutations, demonstrating the existence of a second-site modifier for a specific subset of the phenotypes associated with 22q11DS.

## Author Summary

A range of structural malformations is associated with 22q11 deletion syndrome (22q11DS), which is usually associated with microdeletions at chromosome 22q11.2. Variable defects in cardiovascular, pharyngeal, and craniofacial structures occur, but the basis for such variability is unknown. Mutations in *TBX1*, a gene within the region typically deleted, can recapitulate the structural anomalies of 22q11.2 deletions. However, even among family members with a single *TBX1* point mutation, craniofacial defects are variable. In theory, such differences could depend on an unknown, second-site lesion that modifies the craniofacial consequences of *TBX1* deficiency. We identify such a locus in mouse. In certain strains, lack of the *chordin* gene results in a phenotype resembling severe 22q11DS, also seen in mice lacking *Tbx1*. We find that the *chordin* phenotype depends on a closely linked, strong modifier—a cryptic, partial-function mutation in *Tbx1*. Without it, lack of *chordin* sometimes results in mandibular truncations but no cardiac or thoracic organ malformations. This novel *Tbx1* allele per se results in defects resembling 22q11DS, but with a low frequency of hallmark craniofacial malformations, unless *chordin* is mutant. Thus *chordin* is a modifier for the craniofacial anomalies of *Tbx1* mutations, demonstrating the existence of a second-site modifier for a specific subset of phenotypes associated with 22q11DS.

## Introduction

In approximately 1 in 4000 human births, syndromic congenital malformations are associated with deletions in chromosomal region 22q11.2. DiGeorge syndrome (OMIM 188400), velocardiofacial syndrome (OMIM 192430), and related syndromes are all associated with this deletion; these are collectively called the 22q11 deletion syndromes, 22q11DS [1]. At least 20 genes are contained within the region typically deleted, the DiGeorge Critical Region (DCR).

To understand the roles of particular DCR genes in the etiology of 22q11DS, the functions of many of these genes have been assessed in the mouse. *Tbx1* null homozygotes show severe defects in all the structures variably affected in these syndromes [2],[3],[4], while heterozygotes show aortic arch artery defects similar to some mildly affected patients [2]. Several different point mutations in *TBX1* have been identified in patients with 22q11DS but without cytological deletions at 22q11 [5],[6]. Thus, defective *TBX1* function is a key factor in the pathogenesis of the 22q11DS malformations.

Despite the identification of the DCR, and the key role of *TBX1* in particular, the genetics of 22q11DS pathogenesis remains unclear. Significant numbers of 22q11DS patients don't possess deletions at 22q11.2 or known*TBX1* point mutations, while deletions in other regions of the genome have been observed [7],[8]. These and related considerations suggest that other loci, as yet unknown, play an important role in the etiology of 22q11DS [9]. In mouse models, mutations in *Crkl*, which lies in 22q11.2, and in *Fgf8*, which is unlinked, have both been shown to modulate the developmental phenotype by enhancing the effects of a *Tbx1* null mutation [10],[11]. However, none of the genetic results to date suggest an explanation for the high variability of disease symptoms among 22q11DS patients, or why subsets of patients present with particular structural malformations but not others.

Mice lacking chordin (Chrd), a dedicated antagonist of Bone Morphogenetic Proteins (BMPs), have been reported to show a phenotype recapitulating many structural features of 22q11DS, and very similar to that of *Tbx1* null embryos [12],[13]. Especially given that both genes are expressed in or around the pharyngeal endoderm during early organogenesis, this implied a mechanistic link between BMP antagonism and *Tbx1* function. Consistent with this, reduced levels of *Tbx1* message were observed in the pharyngeal region of *Chrd^−/−^* embryos; moreover, *Chrd* transcript injected into early Xenopus embryos could increase the endogenous *Tbx1* transcriptional level [12]. Taken together, these results suggested that chordin acts in the pharyngeal region to protect *Tbx1* expression from inhibition by local BMPs. However, as detailed below, our breeding of *Chrd* in different wildtype genetic backgrounds has indicated that in most cases, embryos can lack *Chrd* entirely, yet exhibit no resemblance to 22q11DS or *Tbx1* mutants. Such results reveal that an additional, unknown genetic mechanism was at play in the generation of these phenotypes. Here we report our elucidation of the relationship between *Chrd*, *Tbx1*, and the 22q11DS phenotypes.

## Results

**Figure 1:**
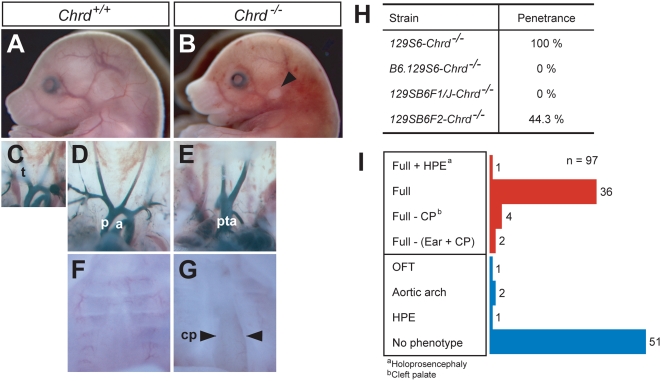
Phenotype of *Chrd* mutant embryos in 129S6 inbred and hybrid backgrounds. (A–G) Comparison of organ structures in wildtype and *Chrd* mutant embryos at embryonic day (E) 15.5. Ears of *Chrd^−/−^* embryos are abnormally located and fail to form auricle structures (A, B). Athymia, persistent truncus arteriosus (PTA; failure of outflow tract septation) and abnormal aortic arch artery structure are observed in the mutant (C–E). Cleft palate (CP) is also a feature of the mutant embryos (F, G). (H) Penetrance of *Chrd* null mutation in various genetic backgrounds; it shows complete penetrance in the 129S6 strain and partial penetrance in the F2 hybrid (129SB6F2) strain. (I) Phenotypic pattern of 129SB6F2-*Chrd^−/−^* embryos. The majority of embryos showed either a ‘Full phenotype’ (as defined in the main text) or no phenotype. Several embryos (11/97) showed variable DGS-like phenotypes. Despite this variability, the embryos can be classified into two groups: those that retain DGS-like phenotypes (in red), and those devoid of DGS-like phenotypes (in blue). a, aorta; cp, cleft palate; HPE, holoprosencephaly; p, pulmonary trunk; pta, persistent truncus arteriosus; t, thymus.

The previously reported *Chrd^−/−^* null phenotype is a completely penetrant constellation of defects – which we call hereafter the ‘full phenotype’ – that includes dysmorphic ear (Figure 1A, B), absence of thymus (athymia), persistent truncus arteriosus (PTA), abnormal aortic arch artery structure (Figure 1C–E), cleft palate (Figure 1F–G). This spectrum of defects is virtually identical to those of *Tbx1* null homozygotes (see Figure S1, available online).

However, *Chrd* phenotypes are highly dependent on genetic background. We observed that *Chrd^−/−^* embryos in an inbred 129S6/SvEv (129S6) genetic background displayed 100% penetrance of this full phenotype. In contrast, mutant embryos in C57B6/J (B6) and random outbred genetic backgrounds were viable and lacked all but a low penetrance of mild craniofacial phenotypes, primarily involving the mandible. This suggested the existence of a strain-specific modifier. To assess this, we interbred 129S6- and B6.129-*Chrd^+/−^* animals, generating F1 and F2 hybrid *Chrd^−/−^* embryos. We detected no phenotype in F1 *Chrd^−/−^* embryos, while F2 *Chrd^−/−^* embryos showed a 44.3% penetrance of phenotypes (43/97; Figure 1H), suggesting either a 129S6-derived recessive modifier or B6-derived dominant suppressor. Because random outbred *Chrd* homozygotes lacked DGS-like phenotypes, the former seemed more likely. The F2 hybrid *Chrd^−/−^* embryos showed a range of phenotypes, suggesting that the effect of a single modifier is incompletely penetrant, or that more than one modifier is present (Figure 1I and Figure S2).

**Figure 2:**
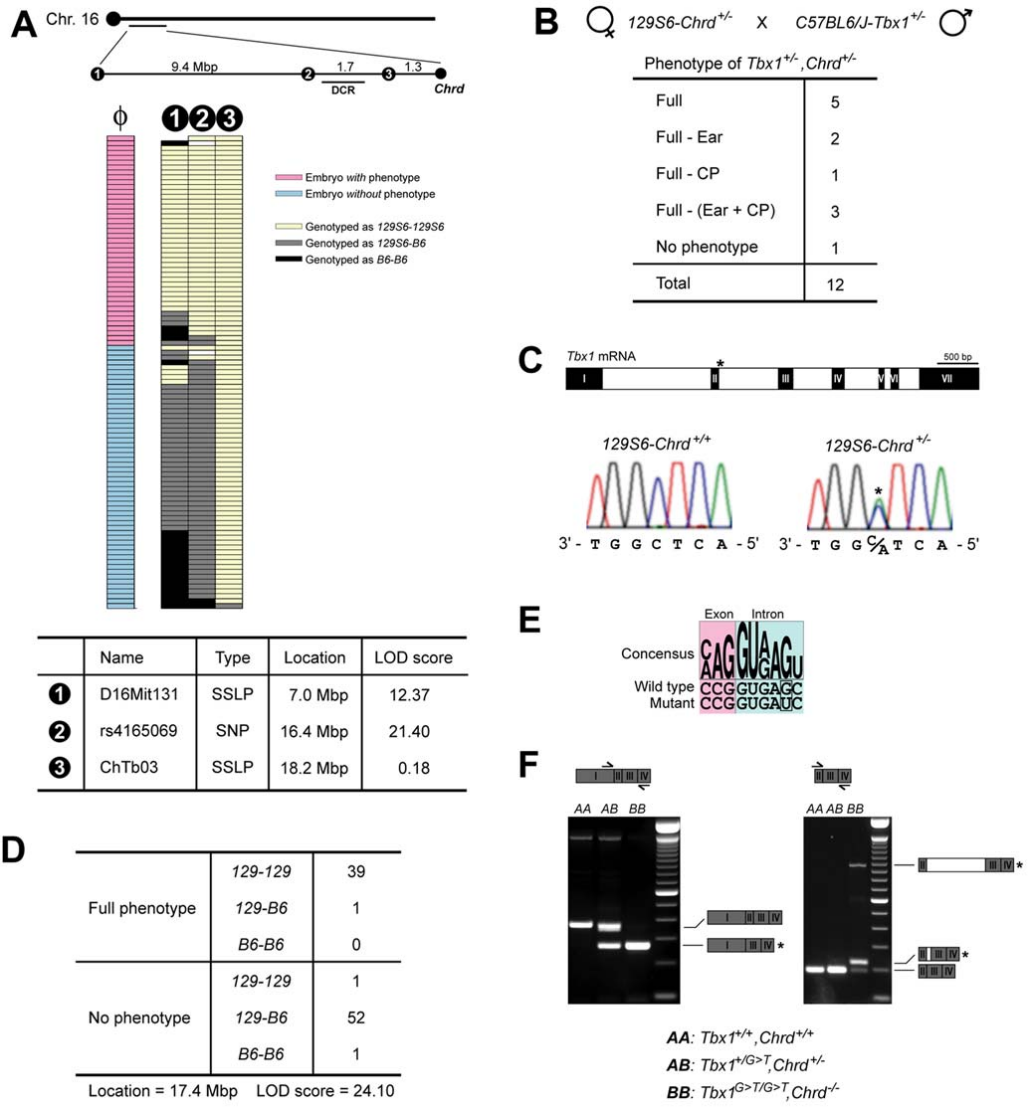
Characterization of a linked modifier of *Chrd* in the 129S6 strain. (A) Schematic diagram of mouse chromosome 16, showing three genetic markers used and genotyping results of F2 hybrid DNAs using these markers. Information on the markers is shown in the table at the bottom of the panel. The second marker rs4165069 shows the strongest co-segregation with the trait. The novel SSLP marker ‘ChTb03’ produces a 271 bp long PCR fragment from 129S6, and 262 bp from B6 (Figure S4). (B) Cross between 129S6-*Chrd^+/−^* and B6-*Tbx1^+/−^* mice produced *Chrd^+/−^,Tbx1^+/−^* embryos with highly penetrant 22q11DS phenotypes, supporting the existence of modifier linked to *Chrd* and suggesting the possibility of a mutation in *Tbx1* itself. (C) Sequencing of the*Tbx1* locus of 129S6-*Chrd^+/+^* and 129S6-*Chrd^+/−^* strains, revealing a specific point mutation (*Tbx1^G>T^*) located in the second intron of the 129S6-*Chrd^+/−^* allele (asterisk). (D) Genotyping results of F2 hybrid DNAs using the*Tbx1^G>T^* mutation as a SNP marker, showing that it is more strongly linked to the phenotype than rs4165069. (E) *Tbx1^G>T^* is located at an exon-intron boundary. Consensus, wild-type, and mutant sequences encompassing the mutation are displayed. (F) RT-PCR analysis demonstrates that *Tbx1^G>T^* disrupts normal splicing of *Tbx1* in 129S6 mice carrying the *Chrd* null allele. As a result of the point mutation, both exon skipping (left) and intron retention (right) occur in the generation of *Tbx1* mRNA, but very little normal message is produced. Diagrams of mRNA with asterisks (*) denote mutant splicing variants that would invariably produce truncated Tbx1 protein.

To begin locating the putative modifier(s), we conducted a genome-wide scan for segregation of a particular 129S6-derived chromosomal region with the *Chrd* locus to result in the full phenotype. Although there was evidence of more than one potential modifier, the data suggested that such a region resides on the same chromosome as *Chrd* (data not shown). In contrast to the situation in humans, *Chrd* is near the DCR in mice (Figure 2A), reflecting a relative chromosomal translocation between *Chrd* and the DCR in these species. Since lesions within the DCR are implicated in similar phenotypes in mouse models [10],[14], we investigated whether a linked modifier might account for the strain-dependence of the *Chrd* phenotypes.

Using several SSLP and SNP markers on our F2 recombinant genomic DNAs, we located a region of 129S6-derived chromosome segregating with the full *Chrd* phenotype; this region lay close to the marker rs4165069, just proximal to the DCR (Figure 2A). These data imply a physically-linked, recessive lesion specific to the 129S6-derived DCR area that is associated with the full *Chrd^−/−^* phenotype.

Because the null phenotype of *Chrd* in the 129S6 background is essentially identical to that of *Tbx1^−/−^*, the simplest explanation is that this region contains a recessive, loss-of-function mutation affecting *Tbx1* activity. If so, the *Chrd* mutant chromosome and linked modifier(s) should be unable to complement a *Tbx1* null mutation. Accordingly, we crossed 129S6-*Chrd^+/−^* with B6-*Tbx1^tm1Bld/+^* to generate double heterozygotes, with one *Chrd* allele and one*Tbx1* allele being nulls created by gene targeting, in trans on the two cognate chromosomes. Among *Chrd^+/−^,Tbx1^tm1Bld/+^* embryos, 5/12 showed the ‘full phenotype’ and 6/12 showed a partial DGS-like phenotype. This indicates the *Tbx1* null allele interacts strongly with the *Chrd* mutant chromosome (Figure 2B). In contrast, when B6.129-*Chrd^+/−^* was crossed with B6-*Tbx1^tm1Bld/+^* mice, no significant phenotype was observed in double heterozygotes (0/15, two cases of asymmetric thymus were detected). Thus the apparent interaction of the*Tbx1* null locus with *Chrd* depends on a linked sensitizer in the 129S6 background. The simplest explanation is a cryptic mutation in *Tbx1* itself.

Sequencing of *Tbx1* revealed a point mutation (G to T) specific to the 129S6-*Chrd^+/−^* strain (Figure 2C). We did not find any *Tbx1* mutation in B6.129-*Chrd^+/−^* animals or in random outbred *Chrd* mice. Furthermore, we did not detect the mutation in 129S6 wild-type, other 129-substrains, or in the R1 ES cell line used for *Chrd* targeting (Figure S3, [12]). These data suggest that this novel mutation (*Tbx1^G>T^*) occurred spontaneously during initial *Chrd^+/−^* ES cell culture or in the establishment of the 129S6-*Chrd* colony.

Utilizing the *Tbx1^G>T^* mutation as a SNP marker, we addressed how strongly it segregates with the full phenotype. The high LOD score suggests that the *Tbx1* mutation is indeed a strong modifier (Figure 2D). Since the mutation is in the boundary region of the second intron and is predicted to disrupt the splicing factor recognition sequence (ESEfinder 2.0; Figure 2E) [15],[16], we examined splicing of*Tbx1* in 129S6-*Chrd* ^−/−^ embryos. We detected exon skipping and intron retention of *Tbx1* mRNA from such embryos, leaving little of the correct form (Figure 2F). This suggests that the *Tbx1^G>T^* mutation is at least partly responsible for the 22q11DS constellation of phenotypes observed in *Chrd* mutants in certain genetic backgrounds.

Having determined that a *Tbx1* lesion is a linked modifier of *Chrd*, we wondered how we observed 44% penetrance of the 22q11DS phenotypes in F2 *Chrd^−/−^* animals (Figure 1H), instead of the expected value of 25%. Breeding records and retrospective *Tbx1* genotyping indicated that this resulted from using a subset of F1 hybrid males as studs that by chance had most often inherited their mutant *Chrd* allele from the 129S6 background, and thus were also carriers of the tightly linked *Tbx1^G>T^* allele (the expected phenotypic penetrance would be 50% if this were the case for all studs used). This caused a non-random bias in transmitting the *Tbx1^G>T^* allele to F2 hybrid animals. Our F1 and F2 genotyping also revealed that *Chrd^−/−^,Tbx1^+/G>T^* mice (in which one *Chrd* null allele was inherited from the129S6 strain and one from B6) had very few 22q11DS phenotypes (1/53 for F2 animals; Figure 2D).

**Figure 3:**
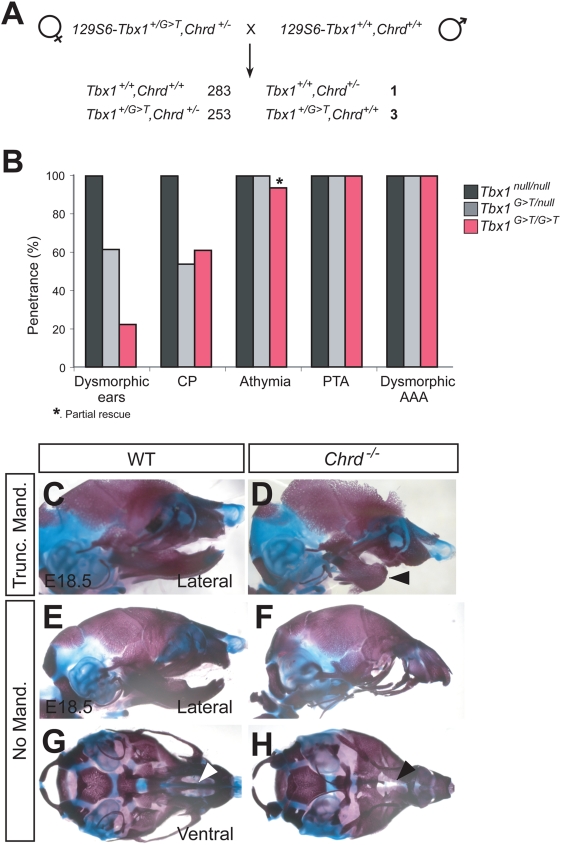
Phenotypes of *Chrd^+/+^,Tbx1^G>T/G>T^* and *Chrd^−/−^,Tbx1^+/+^* mutant embryos. (A) Crossing scheme to generate recombinant animals. 129S6-*Chrd^+/−^* females were mated with 129S6 wild-type studs and offspring were screened for recombination of markers. (B) Penetrance of five phenotypes associated with DGS from each different *Tbx1* mutant class, displaying dosage-dependent rescue of craniofacial phenotypes. (C–H) Variable mandible defects in *Chrd^−/−^* mutant at late gestation stages. (C, D) Mild mandible outgrowth defect in B6-*Chrd^−/−^* mutant embryo (arrowhead). (E–H) Total absence of mandibular elements accompanying incomplete midline structure (arrowhead) is displayed in lateral (E, F) and ventral view (G, H) of 129S6-*Chrd^−/−^* mutant embryo. (I–L) Developmental defects of *Chrd* null, *Tbx1* hypomorphic, and compound mutations. (I) Organs that are defective in various classes of mutant embryos. (J) *Chrd* null mice display a truncated mandible phenotype at low penetrance. (K) *Tbx1* hypomorphic embryos develop very mild craniofacial defects (note partially dysmorphic ear) compared to *Tbx1* null embryos.

To determine the phenotypes resulting from the *Chrd* null or the *Tbx1^G>T^* allele individually, we bred 129S6-*Chrd^+/−^,Tbx1^+/G>T^* with 129S6 wild-type mice to generate recombinant animals (Figure 3A). We genotyped 540 offspring and identified one recombinant carrying only the *Chrd* null allele (*Chrd^+/−^,Tbx1^+/+^*), and three carrying only the *Tbx1^G>T^* allele (*Chrd^+/+^,Tbx1^+/G>T^*).

We assessed the phenotypic consequences of the isolated mutations. Animals heterozygous for the *Tbx1^+/G>T^* mutation are healthy and fully viable, displaying no visible phenotype. We crossed *Tbx1^+/G>T^* animals to themselves and also to *Tbx1^+/null^*. Analysis of the three classes of *Tbx1* mutant embryos for the DGS phenotype demonstrates genetic rescue: *Tbx1^G>T^* homozygotes show rescue of the major craniofacial defects of the *Tbx1* null phenotype (n = 18), while the compound heterozygote (*Tbx1^G>T/null^*) is intermediate (n = 13), relative to the fully penentrant, strong DGS-like phenotype of the null homozygote. Thus *Tbx1^G>T^* is a hypomorphic allele (Figure 3B). That craniofacial development is much less affected in the hypomorphic homozygote relative to the null demonstrates that craniofacial structures are more sensitive to *Tbx1* dose than cardiovascular structures, consistent with a previous report [17].

We also prepared homozygotes for the *Chrd* allele alone, recombined away from *Tbx1^G>T^*. These *Chrd* null homozygotes did not exhibit phenotypes similar to 22q11DS, but showed a low penetrance of variable mandibular truncations, comparable in extent and frequency to what we have observed for B6.129-*Chrd^−/−^* embryos (Figure 3C–H and Table S1). A previous study revealed redundant but essential roles of chordin and noggin, another BMP antagonist, in mandibular outgrowth, during which these BMP antagonists promote cell survival in the developing 1^st^ pharyngeal arch [18]. That study used the outbred *Chrd* strain, which does not carry the *Tbx1* hypomorphic allele. Our study confirms a role for chordin in promoting mandibular development. Interestingly, we also observed a severe mandible truncation in one of the double mutant embryos (*Chrd^−/−^,Tbx1^G>T/G>T^*, Table S1). This result indicates that the craniofacial phenotype caused by loss of *Chrd* is not suppressed by the *Tbx1* mutation, suggesting that *Tbx1* is not in turn a modifier of *Chrd*.

Our data indicating that *Chrd* deficiency is a modifier of *Tbx1* action raises the issue of the molecular basis of this modifier effect. Previous work has suggested that Chrd activity promotes *Tbx1* expression in the pharyngeal region of the mouse embryo. *Tbx1* expression is reduced in this area of *Chrd* null embryos [12]. However, the pharyngeal tissues themselves are deficient from early stages in the *Chrd* mutant (if the cryptic *Tbx1* allele is also present). Nonetheless, ectopic *Chrd* activity in early *Xenopus* embryos can cause increased *Tbx1* transcript levels [12]. In a separate study on the role of BMP signaling in heart development, we observe that BMP-soaked beads in the pharyngeal lumen reduce the expression of *Tbx1* (Choi et al., manuscript in preparation). Such data support a model that *Chrd* expression in the dorsal pharyngeal endoderm promotes *Tbx1* expression in the pharyngeal region, by antagonizing a repressive effect of BMP on *Tbx1* transcription.

**Figure 4:**
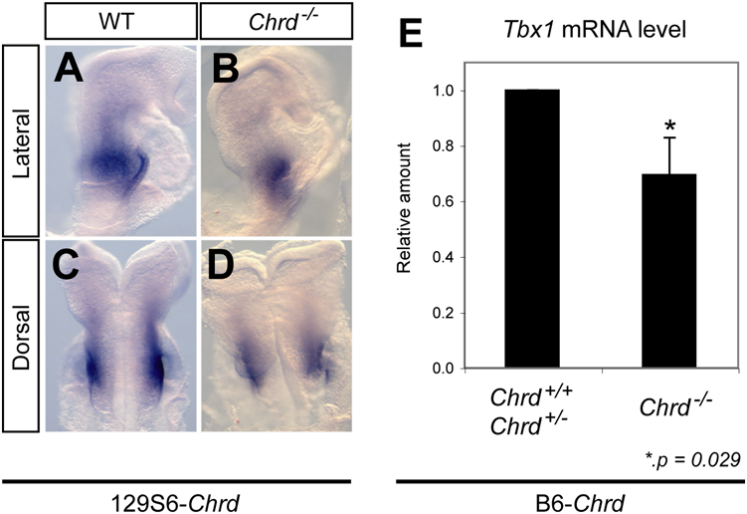
Reduced expression of *Tbx1* in 129S6- and B6-*Chrd^−/−^* embryos. *In situ* hybridization at 5-somite stage of *Tbx1* in 129S6-*Chrd^+/+^* (A,C) and 129S6-*Chrd^−/−^* embryos (B,D), displaying reduced expression in pharyngeal area of homozygotes with a DGS-like phenotype. (E) Quantitative PCR of *Tbx1* mRNA in embryos of the genotypes B6-*Chrd* ^+/+^ and B6-*Chrd* ^+/−^ (n = 8) versus B6-*Chrd^−/−^* (n = 4), demonstrating a modest but significant (p = 0.029) reduction of *Tbx1* expression despite the absence of a DGS-like phenotype in B6-*Chrd^−/−^* mutants.

To further test whether *Chrd* mutations alter *Tbx1* expression in vivo, we measured the amount of *Tbx1* transcripts by *in situ* and real-time quantitative PCR in the 129S6 strain (in the presence of the *Tbx1^G>T^* mutation) and in the B6 strain (without the *Tbx1* mutation). In both cases, there was reduced expression (Figure 4) – about 30% less in B6, despite pharyngeal tissues being fully formed and the *Tbx1* locus presumably wildtype. In sum, our data indicate that *Chrd* has a modest but significant role in promoting *Tbx1* expression. Such a decrease is insufficient to cause 22q11DS-like defects on its own, but may compound the consequences of a *Tbx1* hypomorphic allele.

## Discussion

Our search for the modifier of *Chrd* identified an unexpected, linked mutation in the *Tbx1* locus in the *129S6-Chrd^+/−^* strain, which causes mis-splicing. Comparison of phenotypes between the separated, isolated *Tbx1^G>T/G>T^* and *Chrd^−/−^* mutations and those associated with homozygosity for the original chromososome, i.e. *Chrd^−/−^,Tbx1^G>T/G>T^*, allow us to make important conclusions regarding the basis of the phenotypes we observed. First, we now know that the 22q11DS-like defects seen in the original 129S6-*Chrd^−/−^* mutant embryos were actually caused primarily by the mutation in *Tbx1*. On its own, in both the 129S6 and B6 backgrounds, *Chrd^−/−^* causes a low penetrance of moderate to severe mandibular truncations. Secondly, embryos mutant for the hypomorphic*Tbx1^G>T/G>T^* mis-splicing allele show several cardiac and pharyngeal defects nearly as severe as those of the *Tbx1* null mutants, but without the major craniofacial phenotypes of the null (reduced pinna and cleft palate). Finally, our results show that whereas homozygosity for the hypomorphic *Tbx1^G>T^* allele rarely results in major craniofacial defects alone, it does so when *Chrd* is absent. In contrast, the cardiovascular defects are unaffected. Thus, *Chrd* is a modifier specifically for the craniofacial phenotypes of *Tbx1* lesions.

**Figure 5:**
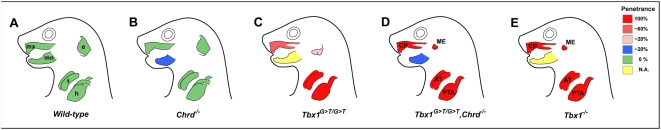
Developmental defects of *Chrd*, *Tbx1*, and compound mutants. Organs that are defective in the various classes of *Chrd* and/or *Tbx1* mutant embryos studied here. Degree of penetrance of phenotypes reminiscent of 22q11DS is shown by variable intensity of red shading, as defined to the upper right of the figure. (A) Normal morphology (indicated by green shading) of organs sensitive to loss of *Chrd* and/or *Tbx1* and their location in wildtype embryos: mx, maxilla; mn, mandible; e, ear; t, thymus; h, heart; CP, cleft palate; ME, malformed ear; AT, athymia; PTA, persistent truncus arteriosus. (B) *Chrd* null mice display a markedly truncated mandible phenotype at low penetrance (blue shading), while other structures are unaffected. (C) *Tbx1^G>T/G>T^* embryos sometimes develop mild craniofacial defects (note partially dysmorphic ear) compared to *Tbx1^−/−^* null embryos, while AT and PTA are fully penetrant. We did not quantitatively score for the presence of previously reported possible subtle mandibular dysmorphology associated with loss of *Tbx1* (yellow, NA: not addressed, [3]), quite distinct from the *Chrd* truncation phenotype. (D) In the *Chrd^−/−^;Tbx1^G>T/G>T^* double mutant embryos, all indicated organs develop abnormally with complete penetrance, with an overall phenotype identical with (E) *Tbx1^−/−^* null embryos. Rarely, these defects are superimposed on mandibular truncations (blue), as seen at similarly low penetrance in *Chrd^−/−^* mutants.

The craniofacial defects associated with homozygosity for the cryptic double mutant reflect a synergistic relationship between the *Chrd* and *Tbx1* lesions. Although there was a low penetrance of striking mandibular outgrowth defects in *Chrd^−/−^* mutants, most homozygotes (73%) looked normal. The *Tbx1^G>T/G>T^* animals in turn showed a limited penetrance of craniofacial defects that are reminiscent of 22q11DS. Penetrance of low-set, reduced pinna (outer ear) was approximately 20%, while cleft palate was about 50%. However, when the two mutations are together, all mutant embryos develop fully penetrant, highly consistent craniofacial phenotypes; these defects appear to be identical to those of *Tbx1^−/−^* null animals, with cleft palate, reduced, low-set ears, athymia, persistant truncus arteriosis, etc (Figure 5). These phenotypes occur against a background of the low-penetrance of severe mandibular truncation defects caused by absence of *Chrd* per se, much more pronounced than the subtle mandibular hypoplasia reported for the Tbx1 null [3]. Therefore, the absence of *Chrd* leads to a synergistic (as opposed to additive) worsening of the defects caused by the hypomorphic *Tbx1* allele, to generate the complete constellation of craniofacial defects seen in the *Tbx1^−/−^* null and reminiscent of 22q11DS.

The *Tbx1^G>T^* mutation we identified behaves according to the genetic definition of a hypomorphic allele: *Tbx1^G>T/G>T^* homozygotes are less severely effected than homozygotes for a null allele, while the compound heterozygote is intermediate in severity. Molecular evidence to account for such reduced activity was apparent in the transcripts produced by the mutant allele. The mutation is predicted to disrupt normal splicing, and in fact we observed via PCR analysis both exon skipping and intron retention. In the latter case, reading frame-shifts result in nonsense codons. The transcript resulting from exon skipping would encode a truncated product lacking 25% of the T-box domain, essential for the proper function of the protein – if any protein is stably produced. In addition, these abnormal transcripts account for most of the spliceforms produced by the allele; we amplified little transcript corresponding to the correctly spliced wildtype version from *Tbx1^G>T/G>T^* homozygotes.

The consequences of the cryptic *Tbx1^G>T/G>T^* allele linked to *Chrd* in the initial 129S6 background account for much of the phenotype of *Chrd^−/−^* mutants as previously reported [12]. Nevertheless, our genomic scan to assess the possibility of modifiers suggested more than one modifier was present. Thus there may be additional modifier(s) in the 129S6 background that influence the *Chrd^−/−^,Tbx1^G>T/G>T^* phenotype. Such lesions could be outside the *Chrd,Tbx1* region, or even within it – the coding regions of these genes are approximately 2 million bases apart (Mouse Genome Informatics). To prove that *only* the *Chrd* targeted mutation and the cryptic *Tbx1* splice site mutation (but no other 129S6 allele) are sufficient for the full phenotype would require reconstituting only these mutations in a different inbred strain, a daunting task. We note that the behaviors of *Chrd* and *Tbx1* alleles independently do not appear to be 129S6-specific. For *Chrd^−/−^* embryos, the penetrance and expressivity of the mandible phenotype in the 129S6 and B6 strains are quite comparable (Table S1). In the case of *Tbx1*, alleles have been made and studied in multiple strains and produced very consistent phenotypes (our observations, [2],[3]). Altogether, it seems likely that if there are additional specific modifiers at play, they have a very minor role.

Previous work and our unpublished observations suggest that Chrd functions to promote *Tbx1* expression in the pharyngeal region. When we assayed *Tbx1* expression in *Chrd* mutants with or without the cryptic *Tbx1* hypomorphic mutation, consistent results were observed. In both 129S6- and B6-*Chrd^−/−^* embryos, there was mild but significant reduction of *Tbx1* expression when compared to the wildtype embryos (Figure 4). Nevertheless, in all three backgrounds tested, *Chrd* null animals free of *Tbx1^G>T^* are viable and show no pharyngeal defects. Thus the decrease of *Tbx1* expression in pure *Chrd* mutants is insufficient to cause a phenotype; however, loss of this activity could be a contributing factor in the functional synergy between the *Chrd* null and *Tbx1^G>T^* mutations in causing a more severe DGS-like phenotype than the hypomorphic *Tbx1^G>T^* mutation alone.

We note that *Chrd^−/−^,Tbx1^+/G>T^* mice very rarely show *Tbx1* mutant phenotypes (1/53 in the F2 hybrids). Thus it is possible that the reduction in functional Tbx1 protein caused by a single allele of *Tbx1^+/G>T^*, compounded by the moderately decreased *Tbx1* expression caused by loss of *Chrd*, is in rare instances sufficient to generate phenotypes similar to 22q11DS.

*Tbx1* also shows genetic interactions with other mutations both within and outside the DCR [10],[11]. However, no second-site mutation has been found previously that can account for why DCR or *Tbx1* mutations are sometimes associated with a particular defect but sometimes not. The result reported here show that *Chrd* is a modifier for the craniofacial anomalies of*Tbx1* mutations, demonstrating the existence of a second-site modifier for a specific subset of the phenotypes associated with 22q11DS.

## Materials and Methods

### Mouse Strains and Genotyping

The *Chrd* null allele (*Chrd^tm1Emdr^*) was generated previously using R1 ES cells [12]. To generate outbred *Chrd* stock [18], germline chimeras were mated to random outbred ICR females, with backcrossing of F1 founders to ICR. To generate 129S6 inbred stock, germline chimeras were mated to 129S6/SvEvTac (Taconic) wild type mice, and *Chrd* heterozygotes backcrossed for >10 generations. To generate B6.129S6-*Chrd*, a 129S6-*Chrd^+/−^* male was crossed to C57BL/6J wild-type (Jackson Laboratory) females. Resulting heterozygous males were subsequently backcrossed with C57BL/6J wild-type females for 10 generations. *Chrd* and *Tbx1^tm1Bld^* were genotyped via PCR as previously described [12]. To type the *Tbx1^G>T^* mutation, regions encompassing the mutation were PCR-amplified (forward: 5′- AGCAGGGCAGGAACAGTCT-3′, reverse: 5′- CTGCCTGGCCAGAGAAGTTA-3′), cut with *DpnII* (New England Biolab), and resolved by agarose gel electrophoresis. For partial genome scanning to find rough general locations of major *Chrd* modifiers, we searched for microsatellite marker differences between 129S6 and C57BL6 strains (http://www.informatics.jax.org/searches/polymorphism_form.shtml; www.cidr.jhmi.edu/mouse/mmset.html). We used 154 markers, with an average interval of approximately 22.2Mb. We designed appropriate PCR primers and amplified corresponding regions from F2 hybrid animals.

### Isolation of RNA and Reverse Transcriptase-PCR (RT-PCR)

To assess *Tbx1* splicing, total RNA was prepared from E9.5 embryos and subjected to RT-PCR. Pharyngeal tissues were homogenized and treated with TRIZOL Reagent (Invitrogen). Remaining tissues were used for genotyping. After further purification, the RNA pellet was dried and resuspended in DEPC-water for subsequent reverse transcription (RT) with random hexamer and mouse mammary tumour virus (MMTV) RT (Invitrogen). Resulting cDNA was used as a template for PCR. The following primers were used for testing *Tbx1* splicing: TbxRT1F 5′-TTTGTGCCCGTAGATGACAA-3′ (forward); TbxRT2R 5′-TCATCCAGCAGGTTATTGGTC-3′ (reverse); TbxRT3R 5′-AATCGGGGCTGATATCTGTG-3′ (reverse); TbxRT4F 5′-TGTGGGACGAGTTCAATCAG-3′ (forward).

### Skeletal Preparation

Skeletal tissues were prepared and visualized as previously described [18].

## Supporting Information

Figure S1 Absence of *Tbx1* results in phenotypes identical to *Chrd* null homozygotes in a 129S6 inbred genetic background. Phenotype of *Tbx1^−/−^* embryo at E16.5 includes abnormal inner and outer ears (A, B), absence of thymus, PTA (C–E) and cleft palate (F,G). a, aorta; cp, cleft palate; p, pulmonary trunk; pta, persistent truncus arteriosus; t, thymus.
(0.3 MB TIF)

Figure S2 Outer ear morphologies in embryos of different *Chrd* genotypes. (A, B) Normal ears of *Chrd^+/+^* and *Chrd^+/−^* embryos at E18.5, respectively. (C–F) Ears of F2 *Chrd* hybrid mutants (129SB6F2-*Chrd^−/−^*) displaying partially-defective outer ear morphologies. (G) Representative ear of inbred *Chrd* mutant embryo, showing the total failure of auricle formation.
(0.9 MB TIF)

Figure S3 Status of *Tbx1* locus in various relevant strains. A number of substrains of 129, the C57BL/6 strain and the parental R1 ES cell line used for targeting are devoid of *Tbx1^G>T^* (represented by +/+genotype). The diagram shows the lineage relationship of many of these strains.
(0.2 MB TIF)

Figure S4 Sequences of novel SSLP locus ChTb03 from B6 and 129S6 strains. Number of ‘CTT’ repetitions differs between the two strains, causing different lengths of amplified PCR product. Bold sequences are primers for PCR amplification.
(0.02 MB DOC)

Table S1 Penetrance of mandible defect phenotype in *Chrd* mutant embryos.
(0.02 MB DOC)
