# Supplementary figures and images for: Chordin Is a Modifier of Tbx1 for the Craniofacial Malformations of 22q11 Deletion Syndrome Phenotypes in Mouse
Source: PLoS Genet. 2009 Feb 27;5(2):e1000395. doi: 10.1371/journal.pgen.1000395 (PMC2640462; doi:10.1371/journal.pgen.1000395)

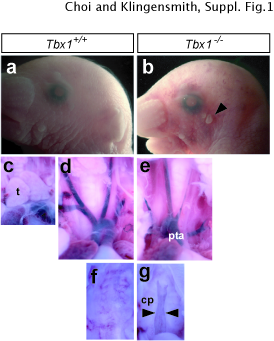

Supplement: Figure S1 — Absence of Tbx1 results in phenotypes identical to Chrd null homozygotes in a 129S6 inbred genetic background. Phenotype of Tbx1−/− embryo at E16.5 includes abnormal inner and outer ears (A, B), absence of thymus, PTA (C–E) and cleft palate (F,G). a, aorta; cp, cleft palate; p, pulmonary trunk; pta, persistent truncus arteriosus; t, thymus. (0.3 MB TIF) [file pgen.1000395.s001.tif]

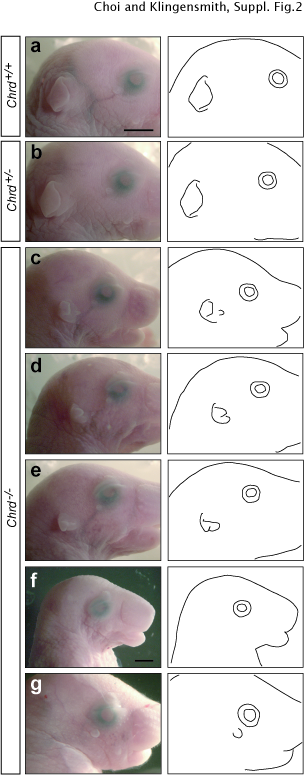

Supplement: Figure S2 — Outer ear morphologies in embryos of different Chrd genotypes. (A, B) Normal ears of Chrd+/+ and Chrd+/− embryos at E18.5, respectively. (C–F) Ears of F2 Chrd hybrid mutants (129SB6F2-Chrd−/−) displaying partially-defective outer ear morphologies. (G) Representative ear of inbred Chrd mutant embryo, showing the total failure of auricle formation. (0.9 MB TIF) [file pgen.1000395.s002.tif]

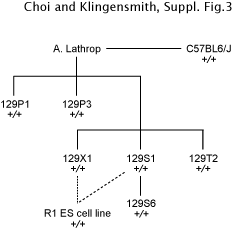

Supplement: Figure S3 — Status of Tbx1 locus in various relevant strains. A number of substrains of 129, the C57BL/6 strain and the parental R1 ES cell line used for targeting are devoid of Tbx1G>T (represented by +/+genotype). The diagram shows the lineage relationship of many of these strains. (0.2 MB TIF) [file pgen.1000395.s003.tif]
