# Supplementary material for: Chordin Is a Modifier of Tbx1 for the Craniofacial Malformations of 22q11 Deletion Syndrome Phenotypes in Mouse
Source: PLoS Genet. 2009 Feb 27;5(2):e1000395. doi: 10.1371/journal.pgen.1000395 (PMC2640462; doi:10.1371/journal.pgen.1000395)
Supplement: Figure S4 — Sequences of novel SSLP locus ChTb03 from B6 and 129S6 strains. Number of ‘CTT’ repetitions differs between the two strains, causing different lengths of amplified PCR product. Bold sequences are primers for PCR amplification. (0.02 MB DOC) [file pgen.1000395.s004.doc]

**Supplementary Fig. 4. Sequences of the novel SSLP locus ChTb03 from 129S6 and C57BL6 strains.**

Bold sequences indicate primers for PCR amplification.

ChTb03-129S6 (271bp)

**TTGCAGCAAAGAAAAGAATTAGG**ATCCAAAGTAATCTTCTTCTTCTTCTTCTTCTTCTTC

TTCTTCTTCTTCTTCTTCTTCTTCTTCTTCTTCTTCTTCTTCTTCTTCTTCTTCTTCTTC

TACTTCTTCTTCTTCTTCTTCTTCTTCTTCTACTTCTTCTTCTTCTTCTTCTTCTTCTTC

TTCTTCTTCTTCTTCTTCTTCTTCTTCTACTACTTCTTCTTCTTTTTTTGTCTGTGATTA

ACGTGTATCAA**ACAGGCTCTTCTGCCAAAAA**

ChTb03-B6 (262bp)

**TTGCAGCAAAGAAAAGAATTAGG**ATCCAAAGTAATCTTCTTCTTCTTCTTCTTCTTCTTC

TTCTTCTTCTTCTTCTTCTTCTTCTTCTTCTTCTTCTTCTTCTTCTTCTTCTTCTTCTTC

TTCTACTTCTTCTTCTTCTTCTTCTTCTTCTTCTACTTCTTCTTCTTCTTCTTCTTCTTC

TTCTTCTTCTTCTTCTTCTTCTACTTCTTCTTCTTTTTTTGTCTGTGATTAACGTGTATC

AA**ACAGGCTCTTCTGCCAAAAA**
