# Supplementary material for: Chordin Is a Modifier of Tbx1 for the Craniofacial Malformations of 22q11 Deletion Syndrome Phenotypes in Mouse
Source: PLoS Genet. 2009 Feb 27;5(2):e1000395. doi: 10.1371/journal.pgen.1000395 (PMC2640462; doi:10.1371/journal.pgen.1000395)
Supplement: Table S1 — Penetrance of mandible defect phenotype in Chrd mutant embryos. (0.02 MB DOC) [file pgen.1000395.s005.doc]

**Supplementary Table. Penetrance of mandible defect phenotype in *Chrd* mutant embryos.**

| Strain | Genotype | Truncated Mandible | No Mandible | Total No. |
| --- | --- | --- | --- | --- |
|  | *Chrd^+/+^* | 0 (0%) | 0 (0%) | 22 |
| 129S6 | *Chrd^+/-^* | 0 (0%) | 0 (0%) | 30 |
|  | *Chrd^-/-^* | 2 (14.3%) | 1 (7.1%) | 14 |
|  | *Chrd+/+* | 0 (0%) | 0 (0%) | 17 |
| B6 | *Chrd+/-* | 0 (0%) | 0 (0%) | 31 |
|  | *Chrd-/-* | 3 (25.0%) | 1 (8.3%) | 12 |
| 129S6 | *Tbx1^+/G>T^,Chrd^+/-^* | 0 (0%) | 0 (0%) | 6 |
|  | *Tbx1^G>T/G>T^,Chrd^-/-^* | 0 (0%) | 1 (14.3%) | 7 |
